# Interactions between gut microbiota and cardiovascular drugs: effects on drug therapeutic effect and side effect

**DOI:** 10.3389/fcvm.2025.1570008

**Published:** 2025-07-10

**Authors:** Tianyu Wang, Qingsu Lan, Haonan Deng, Wenqiang Han, Runtian Zhang, Jingquan Zhong

**Affiliations:** ^1^ Department of Cardiology, Qilu Hospital (Qingdao), Cheeloo College of Medicine, Shandong University, Qingdao, Shandong, China; ^2^ State Key Laboratory for Innovation and Transformation of Luobing Theory, Jinan, China; ^3^ Key Laboratory of Cardiovascular Remodeling and Function Research, Chinese Ministry of Education, Chinese National Health Commission and Chinese Academy of Medical Sciences, Jinan, China; ^4^ Department of Cardiology, Qilu Hospital of Shandong University, Jinan, China; ^5^ Department of Cardiology, Qilu Hospital of Shandong University (Qingdao), Qingdao, China

**Keywords:** gut microbiota, cardiovascular diseases, drugs, aspirin, statin

## Abstract

There exists a complex relationship between gut microbiota and cardiovascular diseases (CVD). On one hand, the plasma levels of various metabolites produced by gut microbiota, such as trimethylamine n-oxide (TMAO), short-chain fatty acid (SFCA), bile acid (BA), are closely related to the occurrence and development of CVD. On the other hand, CVD can affect gut microbiota, leading to gut microbiota dysbiosis or metabolic changes. Cardiovascular drugs are the cornerstone of treating CVD, especially oral medications that play an indispensable role in the long-term treatment of chronic CVD. Increasing research suggests that drugs entering the gastrointestinal environment interact with gut microbiota. Due to the individual differences in gut microbiota, the exploration of its mechanisms is insufficient. Therefore, the purpose of this review is to summarize the interactions between various common cardiovascular drugs and gut microbiota, and to highlight the impact of the gut microbiota on the therapeutical effects and side effects of cardiovascular drugs.

## Introduction

There exists a complex bidirectional interaction between the gut microbiota and drugs, a mechanism that profoundly impacts the efficacy of drug therapies and host health. The gut microbiota can influence drug metabolism and absorption (1–3). The gut microbiota influences drug metabolism through several mechanisms, including the production of enzymes that degrade or activate drugs, altering the pH of the drug absorption environment, and performing biotransformation of drugs (such as demethylation, deamination, dehydroxylation, deacylation, decarboxylation, or oxidation) (4). These processes can significantly impact the efficacy, bioavailability, and pharmacokinetics of various medications. Drugs characterized by low solubility and/or permeability or sustained release are particularly susceptible to these effects, as they tend to have a longer residence time in the gastrointestinal tract. Additionally, the gut microbiota-bile acid axis appears to enhance the solubility of certain low-solubility drugs (5). The influence of the gut microbiota is not confined to the gastrointestinal tract, as certain metabolites derived from it may affect liver function. This occurs by mimicking and competing with intermediates generated during drug metabolism in liver or by influencing the expression of hepatic drug metabolism genes (6, 7).

Conversely, drugs can alter the composition and metabolism of the gut microbiota (8–10). The mechanisms involved include direct or indirect disruption of the gut microbiota, modulation of metabolite production, and changes in the gastrointestinal environment, among others. A few typical examples are worth mentioning. Antibiotics directly disrupt the gut microbiota through their bacteriostatic or bactericidal activities, altering the metabolic capabilities of the microbiota. For instance, ampicillin alters the characteristics of the gut microbiota, reducing its metabolic activity and enhancing the antithrombotic effect of aspirin (11). Aspirin affects the composition of the gut microbiota, reducing the production of mucosal protective metabolites in the intestine, thereby promoting aspirin-induced intestinal injury (12). Proton pump inhibitors (PPI) increase the pH of the gastrointestinal tract, leading to a significant reduction in the *α*-diversity of the intestinal microbiota (13). This promotes the abundance of opportunistic pathogens such as Enterococcaceae and Streptococcaceae, thereby increasing the risk of infections and inflammation. Additionally, PPIs enhance patients’ susceptibility to the pathogenic bacterium *Clostridioides difficile* (14), which is closely associated with numerous cardiovascular diseases (CVD) (15–18). Furthermore, drugs may induce modifications in genes or enzymes involved in their own metabolism or transport, accelerating their own transport and metabolism (19), a phenomenon known as autoinduction. This can have implications for other drugs that share the same metabolic or transport pathways. In summary,these interactions highlight the role of the gut microbiota in drug efficacy and side effects, while also offering potential targets for optimizing therapeutic strategies.

The gut microbiota plays a pivotal role in the pathogenesis and progression of CVD. Key metabolites derived from gut microbiota, such as trimethylamine n-oxide (TMAO), short-chain fatty acids (SCFA), and bile acids (BA), have been extensively studied for their roles in CVD. For instance, TMAO, a metabolite produced by gut bacteria from dietary choline and carnitine, is closely associated with adverse cardiovascular outcomes when its plasma levels are elevated (20). Similarly, SCFA, generated from the fermentation of dietary fibers, exhibit anti-inflammatory and vasoprotective effects (21), while BA are involved in regulating lipid metabolism and energy homeostasis through various signaling pathways (22, 23). Additionally, certain pathogenic bacteria in the gut microbiota, such as *Shigella*, can promote systemic inflammation, thereby increasing the risk of CVD (18, 24–27). Consequently, gut microbiota dysbiosis contributes to the development and exacerbation of CVD. Furthermore, CVD can induce changes in the composition and function of the gut microbiota (28–31), creating a vicious cycle that perpetuates disease progression.

Given the complex interactions between the gut microbiota and drugs, as well as the influence of the gut microbiota on CVD, the gut microbiota also plays a crucial role in shaping the therapeutic effects and side effects of cardiovascular drugs. The gut microbiota exhibits metabolic activity toward many cardiovascular drugs (2, 11, 32, 33) and can influence the transport and absorption of drugs such as aspirin (1), ultimately altering their bioavailability and pharmacokinetics and affecting their therapeutic efficacy. Conversely, cardiovascular drugs can modulate the composition and function of the gut microbiota, thereby influencing their own therapeutic effects and side effects. For example, aspirin alters the composition of the gut microbiota, reducing the production of mucosal protective metabolites and promoting aspirin-induced intestinal injury (12). Nifedipine, with its potential antibacterial activity, inhibits specific gut bacteria, thereby reducing the production of blood pressure-elevating metabolites by the gut microbiota (34). Additionally, different pathological conditions can influence the interactions between the gut microbiota and cardiovascular drugs. For instance, amlodipine increases the proportion of pro-inflammatory bacteria in the gut microbiota of healthy individuals, leading to intestinal inflammation and increased intestinal permeability (35), whereas in individuals with hypertension and non-alcoholic fatty liver disease (NAFLD), it promotes the restoration of intestinal integrity (36). Therefore, exploring the interactions between CVD, cardiovascular drugs and the gut microbiota holds significant implications for individualized drug therapy.

In this review, we will first summarize the associations between the gut microbiota and CVD, aiming to understand the role of gut microbiota and their metabolites in cardiovascular health. We will then explore the mechanisms underlying the interactions between common cardiovascular drugs and the gut microbiota, highlighting the impact of the microbiota on drug efficacy and adverse effects, propose potential therapeutic targets for microbiota-based interventions in cardiovascular medicine.

## Cardiovascular diseases and gut microbiota

The gut microbiota refers to the complex microbial community colonizing the human gastrointestinal tract, comprising bacteria, fungi, viruses, and archaea, with bacteria being the predominant component. The metagenome (collective genetic material) of the gut microbiota far exceeds the human genome in size and establishes a symbiotic relationship with the host, participating in diverse physiological functions such as digestion, immune regulation, metabolic synthesis, and disease defense. Notably, it plays a critical role in maintaining cardiovascular health. Approximately 98% of human gut bacteria belong to the phyla Bacteroidetes, Firmicutes, Proteobacteria, and Actinobacteria. Among these, the ratio of Bacteroidetes to Firmicutes (F/B ratio) is relatively stable under normal conditions, and its dysregulation is associated with metabolic syndromes such as obesity and diabetes. The gut microbiota harbors numerous commensal bacteria, such as *Lactobacillus* species, which produce SCFA that confer cardiovascular benefits, including anti-inflammatory effects, prevention of atrial fibrillation, and reduction of insulin resistance (37–40). *Blautia*, Ruminococcaceae, and *Akkermansia muciniphila* are associated with lower triglyceride levels and exhibit cardioprotective properties (41). *Bacteroides fragilis* has been shown to mitigate high-salt diet-induced hypertension and prevent aging-related atrial fibrillation (42, 43). However, the gut microbiota also includes pathogenic bacteria, such as *Clostridioides difficile*, which significantly increases the risk of myocardial infarction, heart failure, and stroke in infected individuals (15–18). Pathogens like *Shigella*, *Campylobacter*, and *Salmonella* are linked to systemic inflammation and elevated CVD risk (18, 24–27). Certain bacteria exhibit dual roles in cardiovascular health. For example, *Prevotella* produces trimethylamine (TMA, a precursor of TMAO) and is associated with hypertension (44, 45). Conversely, *Prevotella* has also been shown to ameliorate diabetes-induced glucose dysmetabolism and produce SCFA (46–48), suggesting that dietary and other environmental factors may modulate its metabolic activity and impact on host health.

The metabolites of gut microbiota has a complex relationship with CVD. For instance, TMAO is a harmful metabolite associated with adverse cardiovascular outcomes. Meta-analyses have shown a positive dose-dependent association between plasma TMAO concentration and CVD risk (20). TMAO promotes the accumulation of cholesterol in macrophages, leading to their transformation into foam cells, one of the earliest cellular markers of atherosclerosis (49, 50). It also enhances the expression of the nuclear factor-kappa B (NF-*κ*B) pathway and the production of reactive oxygen species (ROS) (51, 52), triggering inflammation and endothelial dysfunction, which contribute to the development of atherosclerosis. Elevated levels of TMAO further promote cardiac hypertrophy and fibrosis through the transforming growth factor-β-mothers against decapentaplegic homolog 2/3 (TGF-β-Smad2/3) signaling pathway, thereby inducing heart failure (53). Additionally, TMAO prolongs the activity of angiotensin II (AngII) by altering its structure, exacerbating hypertension. The gut microbiota also produces metabolites beneficial to cardiovascular health. SCFA, key metabolites derived from the microbial fermentation of dietary fibers, include acetate, propionate, and butyrate. These SCFA exert multiple cardioprotective effects. For instance, acetate and propionate bind to the G protein-coupled receptor 41(GPR41,specific SCFA receptors) on vascular endothelium, regulating vasodilation and reducing blood pressure (54). Acetate downregulates the expression of early growth response-1(Egr-1, a kind of transcription factor) in the heart and kidneys, a critical factor involved in cardiac hypertrophy, cardiorenal fibrosis, and inflammation (21). Butyrate suppresses cholesterol absorption by downregulating Niemann-Pick C1-Like 1 (NPC1L1, key protein for dietary cholesterol absorption) expression (55). Furthermore, BA in the gut are closely linked to metabolism, and their composition and levels are modulated by the gut microbiota (56). BA exert their effects by binding to BA receptors in various tissues. For example, activation of the farnesoid X receptor (FXR) in the liver reduces the expression of lipogenic genes such as sterol regulatory element-binding protein 1c (SREBP-1c), significantly lowering serum and hepatic triglyceride levels (22). Activation of intestinal FXR also reduces lipid levels by decreasing BA reabsorption (23). Takeda G protein-coupled receptor 5(TGR5), another BA receptor, improves insulin sensitivity, reduces inflammation, and ameliorates ventricular remodeling through multiple mechanisms, including modulating DHHC-type palmitoyltransferase 4 (DHHC4), the cyclic GMP-AMP synthase-stimulator of interferon genes (cGAS-STING) pathway, and the cyclic adenosine monophosphate/protein kinase A (cAMP/PKA) pathway (57–59). Activation of TGR5 can also mitigate obesity by stimulating the sympathetic nervous system (60).

On the other hand, CVD can also affect the gut microbiota, leading to gut dysbiosis, which may, in turn, exacerbate cardiovascular conditions. For example, in patients with heart failure, reduced blood flow to the intestinal arteries increases the number of gut bacteria in the mucus layer near the apical surface of the colonic mucosa. This leads to increased permeability of the small and large intestines, allowing higher levels of inflammatory cytokines and endotoxins to enter the bloodstream (61). In addition, the composition of the gut microbiota in patients with heart failure undergoes significant alterations, characterized by a reduction in Coriobacteriaceae, Erysipelotrichaceae, and Ruminococcaceae (62, 34), and an increase in pathogenic bacteria including *Shigella*, *Campylobacter*, *Salmonella*, and *Candida* species (63). A national study in the United States also reported an increase in the number of pathogenic bacteria (such as *Clostridium difficile*) in fecal samples from patients with chronic heart failure, and this pathogenic bacterial infection was significantly associated with increased hospital mortality among heart failure patients (28). Furthermore, in patients with chronic heart failure, downregulation of microbial genes involved in the production of protective metabolites such as butyrate and significant upregulation of intestinal microbes metabolizing harmful metabolites like TMAO and lipopolysaccharide (LPS) have been observed (29). In patients with carotid atherosclerosis, there is an increase in infection-associated gut microbiota, such as *Klebsiella* and *Streptococcus* (30, 31). Patients with hypertension exhibit gene loss in their intestinal microbiome related to amino acid (particularly lysine, histidine, leucine, and serine) biosynthesis and transport, as well as a decrease in fatty acid utilization and carbohydrate transport modules, indicating impaired nutrient synthesis, absorption, and energy production capabilities. In contrast, LPS biosynthesis and export modules are enriched, and LPS has been shown to contribute to inflammation (45). It can be seen that there is an interaction between the gut microbiota and CVD, and this interaction forms a cycle that impacts the health of the organism.

## Interaction between cardiovascular drugs and gut microbiota

### Aspirin

Aspirin, also known as acetylsalicylic acid, is an antiplatelet agent that exerts its therapeutic effects by irreversibly acetylating cyclooxygenase-1 (COX-1), thereby inhibiting the synthesis of thromboxane A2 (TxA2) and suppressing platelet aggregation. This mechanism prevents thrombus formation (64). Compared to its primary metabolite, salicylic acid, the addition of the acetyl group enhances aspirin's water solubility and facilitates its rapid absorption into the bloodstream. Aspirin serves as a cornerstone medication for the treatment and prevention of atherosclerosis. As an antiplatelet agent, enteric-coated tablets are the main form of aspirin, which means it stays in the gut longer and interacts with the gut flora for a longer time.

Aspirin is closely associated with the characteristics of the gut microbiota. Studies have shown that aspirin and its metabolite, salicylate, possess antimicrobial activity. They can reduce cholesterol levels in the cell membranes of certain gut bacteria in a dose-dependent manner, thereby decreasing membrane fluidity. Additionally, aspirin inhibits the activity of dehydrogenase (DHA), a core energy metabolism enzyme that drives redox reactions and ATP production, as well as esterase (EA), a hydrolase involved in lipid metabolism, detoxification, and signal regulation (65). Furthermore, aspirin can induce the lysis of *Helicobacter pylori* (66). The effects of aspirin on gut bacteria are not uniform; they are more pronounced in suppressing the growth of pro-inflammatory and other harmful bacteria, thereby creating a favorable environment for the proliferation of beneficial bacteria. Research has demonstrated that individuals who take oral aspirin exhibit more prominent features of *Prevotella*, *Bacteroides*, Ruminococcaceae, and *Barnesiella* in their gut microbiota compared to control groups. These bacteria are closely linked to cardiovascular health (9). Animal studies have also revealed that aspirin can modulate the composition of the gut microbiota by balancing the ratio of Tregs to Th17 cells and enhancing the cluster of differentiation 39-cluster of differentiation 73 (CD39-CD73) adenosine signaling pathway, which is involved in purinergic signaling. This modulation leads to an increase in the levels of SCFA (67). A comprehensive analysis of the gut metagenomics, host clinical data, and metabolomics of 2,173 European residents found that aspirin improves cardiometabolic health by influencing the gut microbiota. This includes reductions in *Ruminococcus*, *Clostridium citroniae*, and *Parvimonas micra*, lower concentrations of plasma inflammatory markers such as C-reactive protein (CRP) and interleukin-6 (IL-6), and decreased levels of pyruvate (9). These studies collectively suggest that the cardiovascular therapeutic effects of aspirin may be partially mediated by the gut microbiota.

On the other hand, the gut microbiota can also influence the metabolism and absorption of aspirin. Kim et al. found that the gut microbiota metabolizes aspirin into salicylate through esterases produced by certain bacteria. Salicylate, compared to aspirin, is less readily absorbed, thereby reducing aspirin's plasma concentration and weakening its antiplatelet effects. When treated with ampicillin, the abundance of *Enterococci*, *Enterobacteria*, and *Lactobacilli* in the gut microbiota significantly decreased, enhancing the antiplatelet effects of aspirin. This suggests that these bacteria may possess or secrete esterases capable of metabolizing aspirin (11). Additionally, the gut microbiota can influence the absorption of aspirin in the small intestine. Multidrug Resistance Protein 4 (MRP4), an efflux transporter, pumps a variety of structurally diverse endogenous and exogenous organic anions out of cells, and aspirin is also a substrate of MRP4 (68). Studies have shown that the gut microbiota can regulate the expression of MRP4 in intestinal epithelial cells. Jeon et al. demonstrated that treating Caco-2 cells (which structurally and functionally resemble differentiated small intestinal epithelial cells) with coffee bean extract(CBE)-treated fecal microbiota suppressed MRP4 expression, whereas CBE alone did not have this effect. Furthermore, the CBE-treated gut microbiota showed an increase in Muribaculaceae and Lactobacillaceae and a decrease in Proteobacteria, Helicobacteriaceae, and Bacteroidaceae, indicating that these bacteria may play a role in regulating MRP4 expression in intestinal epithelial cells (1).

**Figure 1:**
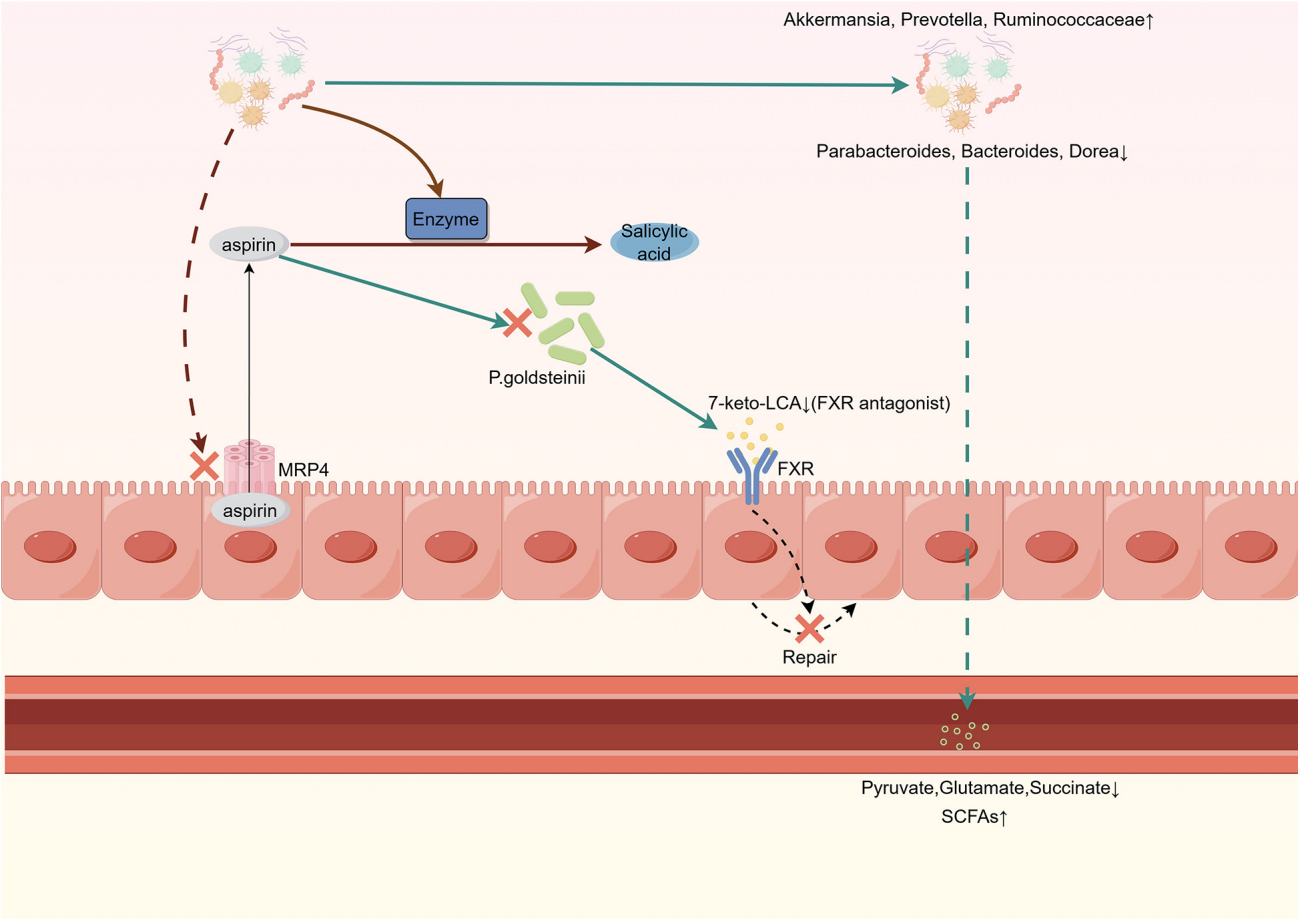
Interaction between gut microbiota and aspirin.Gut microbiota inhibits MRP4 expression, reducing the efflux of aspirin from intestinal cells. Aspirin inhibits *P. goldsteinii*, leading to decreased production of 7-keto-LCA. This weakens its inhibitory effect on FXR, thereby impairing the self-repair ability of intestinal cells. Aspirin increases the abundance of anti-inflammatory bacteria while reducing the levels of pro-inflammatory bacteria in the gut. Gut microbiota metabolic enzymes convert aspirin into salicylic acid, which is less readily absorbed compared to aspirin. MRP4, multidrug resistance protein 4; *P. goldsteinii, Parabacteroides goldsteinii;* 7-keto-LCA, 7-keto-lithocholic acid; FXR, farnesoid X receptor.

Gastrointestinal injury is a common side effect of aspirin, which is traditionally attributed to the inhibition of COX-1 and COX-2. However, recent studies suggest that the gut microbiota may also play a role in gastrointestinal injury caused by aspirin. WU et al. identified *Parabacteroides goldsteinii*, an intestinal microbe inhibited by aspirin. Supplementing with *Parabacteroides goldsteinii* or the BA metabolite 7-keto-lithocholic acid (7-keto-LCA) can promote intestinal epithelial repair by inhibiting FXR receptor signaling, alleviating aspirin-mediated intestinal microenvironment and intestinal barrier damage (12). Through the use of figures, we have provided a more intuitive summary of the interactions between aspirin and the gut microbiota, as detailed in Figure 1.

### Statins

Statins are the most commonly used lipid-lowering drugs in clinical practice, achieving cardiovascular protection by reducing blood lipid levels. Along with aspirin, they are cornerstone medications for the treatment and prevention of atherosclerosis. It is traditionally believed that the antilipidemic effect of statins is mainly achieved by inhibiting 3-hydroxy-3-methylglutaryl-coenzyme A (HMG-CoA) reductase. Additionally, statins are associated with an increased risk of type 2 diabetes mellitus (T2DM), although the mechanism underlying this side effect remains unclear. Recent studies have revealed that the gut microbiota plays a role in both the lipid-lowering effects and the blood glucose-elevating effects of statins. Kari's cross-sectional analysis of cohort studies revealed that the gut microbiota has an additive effect on the risk of statin-associated new-onset type 2 diabetes mellitus (T2DM). Specifically, the abundance of *Ruminococcus torques*, *Blautia obeum*, and *Blautia sp. KLE 1732* was positively correlated with the risk of statin-associated new-onset diabetes (69). Jose et al. discovered that statins activate the liver pregnane x receptor (PXR), which suppresses the expression of cytochrome P450 family 7 subfamily A member 1(CyP7A1, a key rate-limiting enzyme in BA synthesis). This expands the BA pool and alters the proportion of BA, ultimately leading to a deficiency in butyrate-producing bacterial communities and increasing the risk of T2DM (10). Additionally, BA play a significant role in the relationship between statins and the gut microbiota. She et al. found that statins reduce the abundance of *Clostridium* in the gut microbiota, leading to a decrease in the proportion of ursodeoxycholic acid (UDCA) in total BA. UDCA is a ligand for the TGR5 receptor, which stimulates glucagon-like peptide-1(GLP-1) secretion and enhances insulin sensitivity (70). This partially explains the mechanism behind statin-induced elevated blood glucose. In the same study, an increase in the proportion of chenodeoxycholic acid (CDCA) was also observed. CDCA is an endogenous ligand for FXR, and its activation can reduce lipid absorption in intestinal cells and control lipid synthesis in liver cells (23). However, upregulation of liver FXR may attenuate the lipid-lowering effects of statins. He et al. found that FXR upregulation can reverse the inhibition of CyP7A1 induced by a high-fat diet, reducing BA synthesis. Since cholesterol is the precursor for BA synthesis, this may counteract the lipid-lowering effects of statins (3). Furthermore, the gut microbiota metabolizes many drugs, and statins are no exception. *in vitro* studies have shown that simvastatin undergoes bioaccumulation in gut bacteria and is biotransformed by bacterial enzymes (e.g., from *Lactobacillus* and *Bifidobacterium*), leading to delayed absorption and reduced concentration of simvastatin (2). This may involve the off-target effects of statins on the gut microbiota (71), ultimately weakening the lipid-lowering efficacy of simvastatin. Therefore, the regulation of statins’ lipid-lowering effects by the gut microbiota is comprehensive. The individual variability of the gut microbiota may influence its modulation of statins’ effects, which aligns with the observed individual differences in statins’ lipid-lowering efficacy. Future research is needed to further explore the relationship between statins and the gut microbiota, providing more comprehensive strategies for the personalized treatment of statins.

**Figure 2:**
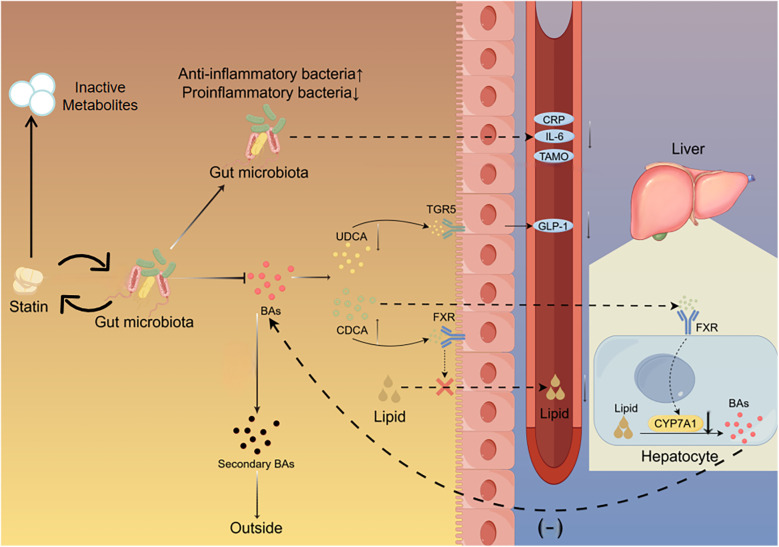
Interaction between gut microbiota and statin. Statins reduce the proportion of pro-inflammatory bacteria in the gut while increasing the proportion of anti-inflammatory bacteria. Statins decrease the proportion of UDCA which binds to the TGR5 on intestinal cells to stimulate the secretion of GLP-1. Statins increase the proportion of CDCA which binds to the FXR in the intestine to inhibit lipid absorption and the FXR in the liver to suppress the expression of Cyp7A1.The gut microbiota metabolizes statins, leading to a decrease in their bioavailability. UDCA, ursodeoxycholic acid; CDCA, chenodeoxycholic acid; TGR5, takeda G protein-coupled receptor 5; FXR, farnesoid X receptor; CyP7A1, cholesterol 7*α*-Hydroxylase; Bas, bile acids.

With further research into statins, it has been discovered that statins may protect the cardiovascular system through mechanisms independent of lipid-lowering, referred to as pleiotropic effects (72). The pleiotropic effects of statins on the cardiovascular system may be related to the gut microbiota. Several studies support the potential of statins to improve gut microbial dysbiosis caused by hyperlipidemia. *Bacteroides2* (Bact2) enterotype is a gut microbiota structure associated with systemic inflammation, and its prevalence is related to body mass index. However, statins can reduce its prevalence (8). Administration of atorvastatin increases the abundance of anti-inflammatory bacteria (*Faecalibacterium prausnitzii*, *Akkermansia muciniphila*, and *Oscillospira*) in the gut microbiota of hyperlipidemia patients, while reducing the abundance of proinflammatory species *Desulfovibrio sp.* and bile-related species (*Bifidobacterium bifidum*) (73). Statins significantly increase the abundance of Bifidobacteria, which are generally considered beneficial, and reduce the abundance of bacteria related to cardiovascular outcomes, such as *Ruminococcus* and *Parabacteroides* (74). Atorvastatin and rosuvastatin significantly increase the abundance of *Bacteroides*, *Butyricimonas*, and *Mucispirillum* species, whose abundances are associated with inflammation (75). Statins are also associated with reduced plasma levels of TMAO (76). Through the use of figures and tables, we have provided a more intuitive summary of the interactions between statins and the gut microbiota, as detailed in Figure 2.

We have summarized clinical trials examining the interactions between aspirin or statins and the gut microbiota (Tables 1, 2), as these two drugs are the most widely used in the treatment of CVD and have been the subject of extensive research.

**Table 1:** Clinical trials of cardiovascular drugs affecting gut microbiota.

Drugs	Number of patients	People characteristics	Changes of gut microbiota	Results
Aspirin (109)	18	Healthy people		↓:TMAO in blood
Aspirin (110)	50	Healthy people	↑:*Akkermansia, Prevotella, Ruminococcaceae*. ↓*:Parabacteroides, Bacteroides,Dorea.*	
Aspirin (111)	2,173	Healthy people and cardiovascular disease Patients	↓:*Neococcus, Clostridium glycyrrhizinilyticum, Micromonas.*	
Statin (8)	888	Obese patients	↑:*Faecalis.* ↓: *Bacteroides*.	
Atorvastatin (112)	20	Colon cancer patients	↑:*Lactobacillus reuteri*.	↑:Tryptophan metabolic derivatives.
Rosuvastatin (113)	66	Healthy people		↑:Betaine, gamma-butylbetaine in blood.
Statin (69)	5,755	Healthy people	↑:*Clostridium salmonellosus*. ↓: *Ysobacterium cellulorum.*	
Statin (74)	143	Acute coronary syndrome patients	↑:*Actinomycete, blautia*, *Bifidobacterium*, anaerobic bacteria. ↓:*Parabacteroides*.	↓:Some metabolites in serum that are associated with disease in blood.

**Table 2:** Clinical trials of gut microbiota affecting cardiovascular drugs.

Measure of change the gut microbiota	Drugs	The number of patients	People characteristics	Effect on the action of drugs
Probiotics (*Bifidobacteriumbreve Bif195*) (114)	Aspirin	66	Healthy people	Reduce the risk of small bowel injury.
Probiotics (*Lactobacillus gasseri*) (115)	Aspirin	64	People treated with aspirin	Gastrointestinal symptoms were reduced, and the small intestinal mucosal rupture and turning red lesions were significantly reduced.
PPI (116)	Aspirin	32	People treated with aspirin	Serum gastrin levels were increased
Vancomycin (117)	Simvastatin	6	Healthy people	No change.
Probiotics (*Lactobacillus casei Zhang*, *Bifidobactetium animalis subsp. lactis V9*, and *Lactobacillus plantarum P-8*) (118)	Atorvastatin	33	Patients with hyperlipidemia	The lipid-lowering effect did not change significantly compared with the control group.
Probiotics (*Clostridium butyricum*) (119)	Rosuvastatin	96	Patients with nonalcoholic fatty liver disease	The lipid-lowering and anti-inflammatory effect was enhanced, Reduces the risk of elevated liver enzymes.

### Ezetimibe

Ezetimibe is a cholesterol absorption inhibitor that reduces blood cholesterol levels by inhibiting the absorption of cholesterol in the small intestine. It has been established that the molecular target of ezetimibe is the sterol carrier NPC1L1, a cholesterol transport protein primarily expressed in the epithelial cells of the small intestine. The gut microbiota is closely linked to NPC1L1, as studies have shown that NPC1L1 gene knockout mice exhibit a decrease in Proteobacteria and an increase in *Bacteroides* when fed a high-fat diet (77). Ezetimibe can similarly induce these changes, along with a reduction in *Desulfovibrio* (78). Notably, an increase in Proteobacteria and a decrease in *Bacteroides* are associated with obesity (79), while a higher abundance of *Desulfovibrio* has been observed in individuals with diabetes (80). Therefore, ezetimibe may regulate the composition of the gut microbiota by inhibiting NPC1L1, ultimately influencing glucose and lipid metabolism. On the other hand, metabolites produced by gut bacteria are also related to NPC1L1 expression. For instance, propionate can increase the number of regulatory T cells and the level of interleukin-10 (IL-10) in the intestinal microenvironment, thereby suppressing NPC1L1 expression in an immune-dependent manner (81). Similarly, inhibiting the production of TMAO can also suppress NPC1L1 expression (82). Thus, dietary interventions, supplementation with SCFA, or the use of TMAO production inhibitors may synergistically enhance the inhibitory effect of ezetimibe on NPC1L1, further strengthening its ability to inhibit cholesterol absorption. However, more research is needed to validate these findings.

### Calcium channel blockers

Calcium channel blockers (CCB) are commonly used antihypertensive drugs in clinical practice, known for their rapid onset of action. They achieve vasodilation by inhibiting calcium channels, thereby reducing the harm of hypertension to the cardiovascular system. The gut microbiota plays a role in the metabolism of CCB. Yoo et al. incubated amlodipine with human and rat feces and observed a gradual decrease in residual amlodipine with increasing incubation time (83). Zimmermann et al. found that diltiazem can be metabolized by *Bacteroides thetaiotaomicron* (32). Zhou et al. found that *Bacteroides dorei* in the gut microbiota of spontaneously hypertensive rat (SHR) was negatively correlated with the maximum concentration and elimination half-life of nifedipine, suggesting that *Bacteroides dorei* may possess enzyme activity capable of directly metabolizing nifedipine. Additionally, serum glycoursodeoxycholic acid (GUDCA) was significantly elevated in SHR, which can upregulate the expression of PXR, leading to a significant increase in the expression of target genes cytochrome P450 family 3 subfamily A member 1 (CyP3A1) and multidrug resistance gene 1a (Mdr1a). CyP3A1 is a key enzyme in drug metabolism, and the P-glycoprotein (P-gp) encoded by Mdr1a limits drug absorption (7). Therefore, the gut microbiota may indirectly reduce the bioavailability of nifedipine by either directly metabolizing it or upregulating hepatic drug-metabolizing enzymes. The question arises: Could improving gut microbiota dysbiosis induced by hypertension be beneficial for increasing the bioavailability of CCB? The answer is likely yes.Studies have shown that under high-altitude hypoxia conditions, the metabolic activity of the gut microbiota weakens, significantly reducing the metabolic rate of nifedipine (84). Direct supplementation with probiotics (85) or antibiotic treatment (83) to alter the composition of the gut microbiota can also increase the bioavailability of CCB.

CCB, in turn, affect the gut microbiota. Many representative drugs of CCB such as amlodipine (86–88), lacidipine (89, 90), felodipine (91), and verapamil (92), have been demonstrated have potential antibacterial activity due to their synergistic or additive effects with many antibiotics.Their chemical structures often contain two benzene rings, and many compounds with biphenyl structures exhibit significant antimicrobial activity, such as quinolone antibiotics. The hydrophobicity of the benzene ring helps the compound penetrate bacterial cell membranes (93), the planarity of the biphenyl structure can promote interaction with bacterial targets (such as enzymes or DNA), inhibiting bacterial DNA replication (94). Studies have shown that amlodipine besylate and amlodipine aspartate can increase the abundance of *Akkermansia*, *Bacteroides*, and *Lactobacillus* in mice with NAFLD and hypertension (36). *Akkermansia muciniphila* is considered a paradigm for the next generation of probiotics, capable of improving insulin resistance, reducing blood lipids, and exerting anti-inflammatory effects (95). *Bacteroides* is generally considered beneficial for host metabolism and immunity in the gut (96). Nifedipine significantly increased the abundance of *Eubacterium* and induced changes in metabolites related to hypertension, such as reduced corticosterone. *Eubacterium* rectale may increase *γ*-aminobutyric acid (GABA) production by regulating amino acid metabolic pathways (97), and GABA can exert antihypertensive effects through central and peripheral mechanisms, including inhibiting sympathetic activity, dilating blood vessels, and promoting sodium excretion (98, 99). Amlodipine can reverse gut microbiota dysbiosis in SHR, restoring the proportion of bacteria that produce lactic acid and acetic acid (100).

Additionally, the side effects of CCB may be related to gut microbiota. Recent studies have proposed that s-amlodipine can cause liver inflammation and dysfunction in rats by affecting gut microbiota rather than liver cells. Through *in vitro* experiments, it was demonstrated that gut microbiota treated by s-amlodipine happened to a proliferation of *Escherichia coli* and a reduction in *Mucispirillum* and *Bacillus uniformis* in the rat intestine, resulting in intestinal inflammation, increased intestinal permeability, and increased production of LPS by intestinal bacteria, which led to an increase in blood LPS levels, causing final liver inflammation and dysfunction (35). However, another study showed that amlodipine has the potential to restore intestinal integrity in NAFLD mice with hypertension, alleviating liver injury and steatosis caused by nonalcoholic fatty liver disease (36). The differences between these two studies may be due to the different models. Therefore, it is necessary to explore the effects of the gut microbiota on CCB under different physiological or pathological conditions.

### Angiotensin-converting enzyme inhibitor/angiotensin II receptor blocker

Angiotensin-converting enzyme inhibitors (ACEI) are compounds that inhibit the activity of angiotensin-converting enzyme (ACE). ACE catalyzes the conversion of Ang I to Ang II, the latter being a potent vasoconstrictor and activator of aldosterone release from the adrenal cortex, significantly elevating blood pressure. Angiotensin II receptor blockers (ARB) selectively block the angiotensin II receptor (AT1 type), thereby inhibiting the effects of Ang II, such as vasoconstriction, increased blood pressure, aldosterone secretion, sodium and water retention, and sympathetic nervous system activation, producing pharmacological effects similar to those of ACEI. Ang II is a key component of the renin-angiotensin-aldosterone system (RAAS) and a major contributor to hypertension and myocardial fibrosis. Recent studies have shown that gut microbiota is involved in the pathophysiological mechanism of AngII in hypertension. AngII affects the α and β diversity of gut microbiota in mice, leading to gut dysbiosis, characterized by an increase in bacteria producing TMAO (101) and a decrease in bacteria producing SCFA (102), both of which have significant impacts on the cardiovascular system. AngII can induce Th17 cells to produce interleukin-17A (IL-17A) which is a key mediator of AngII-induced hypertension and vascular dysfunction (103). Gut microbiota can promote this process because Ang II-induced IL-17A production is attenuated in mice without gut microbiota (104). Can ACEI/ARB reverse the above changes? The answer is possibly yes. Long-term candesartan treatment increases the SCFA level in intestine of SHR (105). SCFA, particularly propionate, can directly act on Th17 cells located in the cecum, reducing IL-17A secretion by inhibiting histone deacetylase (106). Enalapril can improve intestinal permeability and reduce TMAO absorption in SHR. The abundance of *Coprococcus* which is considered a butyrate producer was increased in SHR fed with Enalapril, which is beneficial for lowering blood pressure (107). However, Yang et al. found that *Coprococcus* has esterase activity, which can decompose ester-type ACEI (33), impairing the antihypertensive effect of ACEI. *Lactobacillus* can produce GABA through L-glutamate metabolism. High-salt diet is an important factor for hypertension, which can deplete *Lactobacillus* in mice (108), while candesartan can counteract the decrease of Lactobacillus caused by hypertension, and candesartan treatment also increases the intestinal expression of genes encoding tight junction proteins (such as zonula occludens, occludin, and claudin-1), improving increased intestinal permeability caused by hypertension and preventing gut microbiota translocation (105). These findings indicate that ACEI and ARB exert part of their therapeutic effects through the gut microbiota.Future research is needed to elucidate the molecular mechanisms of the interaction between ACEI/ARB and the gut microbiota.

## Conclusion

There are complex interactions between gut microbiota and cardiovascular drugs. We discussed the main interactions between the two. On the one hand, cardiovascular drugs cause changes in the composition and metabolism of gut microbiota, affecting the production and absorption of metabolites related to cardiovascular health such as TMAO, SCFA and BA. On the other hand, many cardiovascular drugs will be metabolized by gut microbial enzymes due to the ability of gut microbiota to metabolize exogenous substances and the self-induction of drugs. Cardiovascular drugs will not only affect specific intestinal bacteria, but also change the overall intestinal microbial characteristics of people with CVD, making them closer to the intestinal microbial characteristics of healthy people. It has potential impact on the overall health level of the body. Personalized treatment of CVD is promising based on individual differences in the gut microbiota. By evaluating the characteristics of a patient's gut microbiota, more suitable cardiovascular drugs and dosages can be selected for their specific needs, thereby improving treatment effectiveness and reducing side effects. Further research on the mechanism of the interaction between the two is needed to provide new perspectives and new strategies for the treatment of CVD.
